# Periodontal and Dental Status in Packs of Spanish Dogs

**DOI:** 10.3390/ani11041082

**Published:** 2021-04-10

**Authors:** Ana Whyte, Jaime Whyte, Luis V. Monteagudo, Alberto García-Barrios, M. Teresa Tejedor

**Affiliations:** 1 Department of Animal Pathology, Faculty of Veterinary Sciences, University of Zaragoza, C/Miguel Servet 177, 50013 Zaragoza, Spain; awhyte@unizar.es; 2 Department of Human Anatomy and Histology, Faculty of Medicine, University of Zaragoza, C/Domingo Miral s/n, 5009 Zaragoza, Spain; jwhyte@unizar.es (J.W.); agarciab@unizar.es (A.G.-B.); 3 Department of Anatomy, Embriology and Animal Genetics, Faculty of Veterinary Sciences, University of Zaragoza, C/Miguel Servet 177, 50013 Zaragoza, Spain; monteagu@unizar.es; 4 CIBER CV (University of Zaragoza—IIS), Faculty of Veterinary Sciences, University of Zaragoza, C/Miguel Servet 177, 50013 Zaragoza, Spain

**Keywords:** canine, periodontal disease, dental calculus, gingiva

## Abstract

**Simple Summary:**

Periodontal disease (PD) is the most frequently occurring canine oral pathology, and even at early stages, it may significantly affect general health. Therefore, veterinarians should be able to properly diagnose PD, treat PD at earlier stages, and establish preventive strategies. However, data about PD prevalence in different sizes and breeds of dogs are highly variable and incomplete. Our objective was to improve understanding of PD in dogs by studying a specific group of animals that were subjected to specific management: pack dogs in Spain. Thirty-two conscious individuals from two packs of dogs in Northeastern Spain (30/32 crossbred hunting dogs and 2/32 Siberian Husky; 26 males and 6 females; 27.75 ± 5.807 kgs; 5.48 ± 2.818 years; mixed diet: home-prepared food, commercial dry food, stale bread and bones) received visual dental examinations for assessment of absent teeth (AT), dental calculus (DC) grade, gingival recession (GR), periodontal disease (PD), tooth fracture (TF), and dental attrition (DA). The low prevalence (15.62%) and extent of PD (<1 affected tooth/individual) was attributed to diagnosis methodology, bodyweight effect, breed, and, ultimately, diet. Individuals affected by DC remained under veterinary surveillance due to risk of PD development.

**Abstract:**

While periodontal disease (PD) is the most common canine oral pathology, its prevalence varies according to diagnosis methodology, breed, and age. We intended to increase understanding of canine PD by studying dogs that are managed in a specific way: pack dogs in Spain. They received a mixed diet (home-prepared food, commercial dry food, stale bread and bones). Thirty-two conscious individuals from two packs of dogs in Northeastern Spain (30/32 crossbred hunting dogs and 2/32 Siberian Husky; 26 males and 6 females; 27.75 ± 5.807 kgs; 5.48 ± 2.818 years) received visual dental examination for assessment of absent teeth (AT), dental calculus (DC) grade, gingival recession (GR), periodontal disease (PD), tooth fracture (TF), and dental attrition (DA). DC was the most prevalent oral problem (75%), followed by TF/DA (68.75%), AT (34.37%), GR (31.25%), and, finally, PD (15.62%). Low individual affectation values were found for AT, GR, and PD (<1 tooth/individual); mean DC grade per individual was 0.06 ± 0.063; and TF and DA were found in 1.63 and 4.72 teeth/individual, respectively. Low prevalence and extent of PD was attributed to diagnosis methodology, bodyweight effect, breed, and, ultimately, diet. Individuals affected by DC remained under veterinary surveillance due to PD development.

## 1. Introduction

Periodontal disease (PD) is the most frequently detected oral pathology in dogs [1]. PD refers to inflammatory lesions in the periodontium, affecting tissues supporting the tooth (gingiva, cementum, periodontal ligament, and alveolar bone), and is initiated by dental plaque [2]. PD is a progressive disease with two different phases [3,4]: gingivitis, a reversible inflammation of the gingiva, and periodontitis, which is inflammation of tooth support tissues that ultimately leads to attachment loss due to destruction of the periodontal ligament, cementum, and alveolar bone. According to Koch’s postulates, PD cannot be considered as an infection [5]; Socransky modified these postulates to propose a commensal/opportunistic hypothesis to explain the role of bacteria in PD [6,7]. The overgrowth of bacteria in the plaque is controversial as a cause of PD; the host response to these bacteria seems the most likely explanation for the characteristic tissue changes of PD [5]. Several factors influence this individual response [3] and may be considered as risk factors for PD; breed, bodyweight, age, diet, oral care, and chewing habits [4,8,9,10].

PD is a common disease in dogs and one of the main conditions treated by primary care veterinary practices. Prevalence data are highly variable, based on diagnosis methodology (9.3–100% for oral visual examination and detailed examination of anaesthetized dogs, respectively) [11,12], breed (2.4–25.2% for Staffordshire Bull Terrier and Yorkshire Terrier, respectively) [11], and age (approximately 80% of dogs over 3 years of age presented some degree of PD) [13].

Even in early stages, PD may have a significant impact on a dog’s general health [14]. Chronic bronchitis, pulmonary fibrosis, endocardiosis and endocarditis, interstitial nephritis, and hepatitis have been associated with PD [15,16]. Therefore, PD must be treated as a disease capable of threatening the individual’s life and not as a merely dental pathology [17]. For this reason, veterinarians should be able to properly diagnose PD, treat PD at earlier stages, and establish preventive strategies. However, data about PD prevalence and extent in different sizes and breeds of dogs are highly variable and incomplete. Our objective was to improve understanding of PD in dogs by studying a specific group of animals that were subjected to specific management: pack dogs in Spain.

## 2. Materials and Methods

A total of 32 dogs (26 males, 6 females) were evaluated (Table 1). These animals were part of two packs of dogs in Teruel province (Northeastern Spain), located in two zoological nuclei (16 individuals per location). According to current Spanish law, a pack of dogs is a group of twelve or more dogs kept for hunting, guarding, or pulling sleds, who share accommodations (Law 11/2003, 24 November 2003, about Animal Protection) [18]. A pack of dogs must be hosted in a zoological nucleus. These zoological nuclei, when legally registered, are engaged in animal welfare and meet current legal requirements [18]. No other animal, domestic or wild, was housed together with these dogs in the two zoological nuclei considered. These zoological nuclei were not rescue kennels and the dogs belonged to the nuclei owners, who trained them periodically. When the dogs were not working or training, they were housed in individual kennels (1.60 × 2.75 × 1.86 m; 4.4 m^2^) provided with cement floors, roofs, and mesh enclosures (galvanized material).

The only inclusion criterion was an age of ≥1 year. Most individuals were crossbred hunting dogs (30/32; 93.7%) and 2/32 (6.3%) were Siberian Huskies (Table 1). Their ages ranged from 1 to 11 years (median: 6 years; mean ± SD: 5.48 ± 2.818 years). All dogs were medium-large or large, with weights ranging from 14 to 40 kg (median: 28.5 kgs, mean ± SD: 27.75 ± 5.807 kgs; Table 2).

All dogs received a basic diet consisting of home-prepared food (cooked meat, potatoes, and rice), stale bread, and commercial dry food. The diet was supplemented with jam bones and raw meat in one of the zoological centers and with jam rinds and bones from other species in the other zoological center. Neither brushing of the teeth nor dental hygiene chews were applied. The dogs considered have been in these nuclei all their lives (either they were born in it or were bought as puppies by their owners), being fed the described diet daily.

Ethical review and approval were waived for this study since the data were recorded in the course of a routine veterinary check-up. Spanish law for animal protection and non-experimental veterinary procedures [19] was followed in this work. An informed consent form was signed voluntarily by the owner for inclusion of the animals in the study and publishing any results obtained.

Only visual dental examinations were performed on conscious dogs to assess absent teeth (AT), the extent of dental calculus (DC), gingival recession (GR), periodontal disease (PD), tooth fracture (TF), and dental attrition (DA). Labial and buccal surfaces of the teeth and gingival margins were examined, once the lips and cheeks were retracted. For correct examination of both mandibular teeth and gingiva, the mouth was carefully opened. Since no dental probe was used, the Weighted Gingivitis or Periodontitis Score System (WGS, WPS) could not be applied [20]. Data from each tooth were recorded in an individual data sheet, including a dental chart.

DC was assessed as follows: 0 (no calculus); 1(calculus coverage of crown: 1–25%); 2 (calculus coverage of crown: 25–50%); 3 (calculus coverage of crown: 50–75%); 4 (calculus coverage of crown: 75–100%) [21].

The global state for DC of each individual mouth was assessed using a calculus global index for the animal (mean DC grade per individual):

Calculus global index: (n_0_x_0_ + n_1_x_1_ + n_2_x_2_ + n_3_x_3_ + n_4_x_4_) / (n_0_ + n_1_ + n_2_ + n_3_ + n_4_) (1)

where n_0_, n_1_, n_2,_ n_3_, and n_4_ represent the number of teeth assessed with calculus grades 0, 1, 2, 3, and 4, respectively (x_0_, x_1_, x_2_, x_3_, and x_4_), for each dog. Therefore, the calculus global index is a continuous variable, ranging from 0 (no tooth affected by calculus) to 4 (all teeth in calculus grade 4).

Only presence/absence per tooth was recorded for GR, PD, TF, and DA. GR was considered present if the distance from cementum–enamel junction to the gingival margin appeared visually extended, without any sign of dental calculus, nor tissues affectation. PD diagnosis was carried out in accordance with recommendations from the American Veterinary Dental College [22]; gingivitis (stage 1), and any degree of periodontitis and attachment loss (stages 2–4), were considered to indicate the presence of PD, while clinically normal aspects of the tooth indicated absence of PD (stage 0) [23]. Type of TF was also recorded: complicated crown fracture (type 4) or not complicated crown fracture (type 3); type 4 fractures involve enamel and dentin with pulp exposure, while pulps are not exposed in type 3 fractures [22].

The statistical analysis was carried out by using IBM SPSS Statistic 22.0 software; statistical tests were conducted and interpreted in accordance with the recommendations of Petrie and Watson [24]. Relationships between age and number of affected teeth and age and calculus global index were assessed using Spearman´s Rank correlation coefficients (ρ, nonparametric tests). Pearson χ^2^ test was used to compare the frequencies of the considered anomalies/diseases among types of teeth. *p* values < 0.05 were considered statistically significant.

## 3. Results

Table 1 shows the oral health characteristics of the studied animals. Only 6/32 individuals (18.75%) showed no process at all (no AT, DC, GR, PD, TF, or DA). Either alone or in conjunction with other anomalies, DC was detected in 24/32 dogs (75%), AT were found in 11/32 dogs (34.37%), and GR was present in 10/32 dogs (31.25%). PD was detected in 5/32 individuals (15.62%), usually in the presence of the rest of processes (4/5; 80%), with one exception, where only DC, GR, TF, and DA were present (1/5; 20%). On the other hand, most individuals showed TF and/or DA (22/32; 68.75%).

The results obtained per individual for AT, calculus global index, GR, PD, TF, and DA are shown in Table 2. The most frequent anomaly was DA (4.72 ± 6.223 DA-affected teeth/individual), followed by TF (1.63 ± 1.897 TF-affected teeth/individual). GR, AT, and PD were rare (on average, less than one affected tooth per individual). Calculus global index values were also low (0.06 ± 0.063). Positive significant correlations were detected for age and AT per individual (0.402; *p* = 0.028), DA-affected teeth per individual (0.551; *p* = 0.002), and FT per individual (0.414; *p* = 0.023). A single individual, aged 10 years, showed both maximum DA and GR (21 and 6 affected teeth, respectively).

Table 3 summarizes the results obtained by type of tooth. The permanent canine dental formula is as follows: 2 (incisor (I) 3/3, canine (C) 1/1, premolar (PM) 4/4, molar (M) 2/3). In total, 23/1344 teeth (1.7%) were absent; both incisors and premolars showed the highest frequency of absent teeth (2.9 and 2.1%, respectively), while only one canine was absent, and every molar was present. The causes of these absences are unknown; individual dental histories were not available, and radiographs were not performed.

As shown in Table 3, DC was detected in 5.5% of present teeth (73/1321). Most teeth with calculus showed grade 1 (69/1321; 5.3%), while both grades 2 and 3 were detected in only two teeth (one molar and one premolar, in both cases). Canines were the teeth most affected by DC (54/127; 42.5%), even though only grade 1 was detected. Frequency of DC was significantly lower for incisors, premolars, and molars (1.1%, 1.0%, and 3.1%, respectively (*p* < 0.05)); only grade 1 was detected in incisors, but all grades were detected in molars and premolars, with low frequencies for both grades 2 and 3, as previously noted.

GR was detected only in 27/1321 (2% of present teeth). As shown in Table 3, the highest frequency of GR corresponded to incisors (18/373; 4.8%) and significantly differed from the lowest frequency, detected in premolars (3/501; 0.6%; *p* < 0.05). Frequencies in canines (1/127; 0.8%) and molars (5/320; 1.6%) were low and did not differ from the rest of frequencies.

Only 6/1321 (0.5% of present teeth) showed PD. Canines did not show PD and PD were present in very low frequencies in other types of teeth; no significant differences were detected among types of teeth (*p* > 0.05, see Table 3).

DA was the most frequent anomaly in the studied individuals; 11.4% of present teeth showed DA (151/1321). As shown in Table 3, the frequency of DA in incisors (117/373; 31.4%) was significantly higher than in the rest of the teeth (*p* < 0.05).

TF was detected in 52/1321 (3.9% of present teeth). Canines showed the highest frequency of fractures (21/127; 16.5%), which significantly differed for the rest of the teeth (*p* < 0.05, see Table 3). Globally, both fracture types (both types 3 and 4) were almost equally frequent (1.9% and 2%, respectively); however, compared to other types of teeth, a majority of both type 3 and type 4 fractures were observed in canines (12.6% and 3.9%, respectively).

## 4. Discussion

AT were frequently detected (in 34.37% of the studied dogs), although statistical values for AT/individual showed low individual affectation (see Table 2). AT can be caused by attachment loss due to PD [2], but other causes are impact on teeth, congenital loss, extraction for TF, or other causes not related with PD. No dental history was available, and no radiographs were performed; therefore, the causes of these absences were unknown. However, fractures and attrition could be considered traumatic predisposition, causing AT, since TF and/or DA were also found frequently (68.75%). Statistical values for TF and DA/individual showed the highest individual affectation (see Table 2). Age might also explain the maximum values observed for AT/individual, TF/individual, and DA-affected teeth/individual, since correlation values for age and these variables pointed to a moderate strength of these age associations [25].

Previous studies reported incisors, premolars, and molars as the teeth most frequently absent in dogs, possibly due to PD [26]. In the present study, incisors and premolars were the most frequently absent teeth, while no molar was absent. AT could be due to causes other than PD; in fact, DA frequency in incisors was significantly higher than in other types of teeth (*p* < 0.05), although the frequency of TF was similar for both incisors and premolars, and significantly lower than for canines.

DC results from the mineralization of dental plaque, the porous surface of which facilitates adherence of new plaque [27]. Bacteria in the plaque cause an immune response resulting in inflammation and even tissue damage [8]. DC was detected in 75% of studied dogs, although statistical values for calculus global index/individual showed low individual affectation (see Table 2); however, in view of the important role that DC can play in the development of PD, periodic dental surveillance of these animals would be advisable. Harvey et al. [28] found that DC was most extensive on premolars and molars in a large-scale study on numerous purebreds and mixed-breed dogs. Similar results were found in the present study; DC in grades 2 and 3 was only found in premolars and molars, but in low frequencies.

Redness, inflammation, receding, or hyperplasia of gingiva in the presence of dental calculus are recognized signs of PD in visual dental examinations [29]. However, previous studies showed that GR could occur in the absence of plaque and inflammatory cells [30]. Other affectations, such as malocclusion, mechanical overloads, or dental trauma, could also cause GR without any sings of PD [30]. As mentioned in the Material and Methods section, we considered GR apart from PD, the absence of dental calculus and tissue affectations being their differential characteristics. Considering this diagnosis, GR was detected in 31.25% of dogs, although statistical values of GR/individual showed low individual affectation (see Table 2). In the present study, the highest frequency of GR was associated with the areas around the incisors, but the lowest frequency was detected around premolars (*p* < 0.05). In humans, GR was more frequently found in buccal surfaces [31] and in monoradicular teeth (incisors and canines) than in molars [32]; these results agree with those obtained in the present study. A mechanical origin could explain for this frequent GR not being related to PD, affecting the incisors, especially; the diet and lifestyle of the studied dogs that use their mouths to collect prey, and usually bite the mesh enclosure of their kennel, could cause the observed GR cases.

Visual examination of a conscious dog´s mouth has several limitations for PD detection [33]. Examination of all surfaces of the teeth is not possible. Full assessment of tissue loss around the teeth requires clinical probing and intraoral radiographs. Reported PD prevalence from visual examination of conscious dogs is between 9.3% [11] and 18.2% [34]; data from anaesthetized animals or from necropsy show PD prevalence between 44% [35] and 100% [12]. PD prevalence found in the present work (15.62%) was within the range previously reported for visual examination of conscious dogs. The prevalence of PD in the studied individuals is likely to be underestimated. Visual assessment of PD in non-anesthetized dogs showed moderate agreement with the reference standard diagnosis, based on use of periodontal probing and dental radiography in anesthetized animals (41.57% of agreement and weighted kappa = 0.42, 95% confidence interval: 0.29–0.55) [29]. Additionally, when experienced dentists perform these visual assessments, substantial inter-examiner reliability is achieved (61.02% of agreement and weighted kappa = 0.63, 95% confidence interval: 0.49–0.76) [29]. Therefore, visual assessment could be useful to detect animals needing dental care, when a larger number of dogs must be examined out of clinic facilities.

Several studies showed variations of PD prevalence among breeds and pointed to a bodyweight effect. Harvey et al. [28] found a significant and negative correlation for body weight, and both the gingival index and attachment loss. Butković et al. [36] observed the highest level of PD in medium-size breeds (39.5%), followed by large breeds (26.3%), small breeds (20.0%), and mongrels (14.0%). O’Neill et al. [11] reported the highest PD prevalence in Yorkshire Terriers (25.2%), followed by Cocker Spaniels (12.0%), Jack Russell Terriers (9.5%), Border Collies (6.7%), German Shepherds (4.5%), Labrador Retrievers (3.2%), and finally, Staffordshire Bull Terriers (2.4%). The observed PD prevalence (15.62%), probably underestimated by the diagnosis method, was in the interval between large breeds and mongrels (26.3–14%), described by Butković et al. [36], and corresponded to the large size of the mostly crossbred dogs in the studied sample.

Statistical values for PD/individual showed low individual affectation (see Table 2) and only 0.5% of present teeth showed PD (6/1321, see Table 3). Prevalence seems to be related to bodyweight; PD was assessed in 28% of teeth in Miniature Schnauzers [37], 30.5% of teeth in Yorkshire Terriers [38], and only 6% of teeth in Labrador Retrievers [34].

In dogs, several studies pointed to premolars and molars as the teeth most frequently affected by PD [12,35], while more recently, the highest risk of PD was detected in incisors [33]. In the present study, no significant differences among teeth were found for PD, but frequency of PD was highest in molars, followed by premolars and incisors, with no PD cases in canines. These different results might be explained by differences in diagnosis methodology, but there also seems to be an effect as a result of breed [34,37,38].

It is generally accepted that PD becomes more frequent and serious with increased age [34,37,38]. In the studied animals, both mean (5.48 years) and median (6 years) ages were not very high, and this could explain the low observed values for PD prevalence and individual affectation.

The effect of diet consistency on PD incidence and severity is controversial in dogs. While Watson [39] recommended harder foods requiring vigorous prehension and mastication, other authors did not find evidence of a relationship between diet consistency and PD incidence [12,40]. More recently, Gawor et al. [41] suggested that soft diets could increase the likelihood of PD by increasing accumulation of dental plaque and calculus. Buckley et al. [42] reported a significant beneficial effect of feeding only commercial pet food compared with the home-prepared diet (soft diet) when at least part of the diet was composed of dry pet food. Daily consumption of dental treats was also effective in improving dental health, according to Buckley et al. [42]. The use of appropriate chew toys could reduce plaque and calculus [43]. The studied dogs received both home-prepared food and commercial dry food together with stale bread and bones, which, due to their abrasive character, could play the role of chew toys; this kind of mixed diet could explain both low levels of PD and TF/DA occurrence.

## 5. Conclusions

In the studied population, DC was the most prevalent oral health problem (75%), followed by FT/DA (68.75%), AT (34.37%), GR (31.25%), and, finally, PD (15.62%). The low prevalence and extent of PD could be due to diagnosis methodology, bodyweight effect, breed, and, ultimately, diet. However, the results of this work have value as an initial PD assessment. On the other hand, because of PD pathogeny, animals affected by DC should be under veterinary surveillance due to their risk for developing PD.

## Figures and Tables

**Table 1:** Characteristics of studied animals.

Variable	Frequency
Males	26/32 (81.3%)
Breed	
Crossbred hunting dog	30/32 (93.7%)
Siberian Husky	2/32 (6.3%)
No process	6/32 (18.75%)
Absent teeth, dental calculus, gingival recession, periodontal disease, tooth fracture, and dental attrition	4/32 (12.5%)
Absent teeth and dental calculus	1/32 (3.1%)
Absent teeth, dental calculus, and dental attrition	1/32 (3.1%)
Absent teeth, dental calculus, tooth fracture, and dental attrition	2/32 (6.2%)
Absent teeth, dental calculus, gingival recession, tooth fracture, and dental attrition	3/32 (9.3%)
Dental calculus	3/32 (9.3%)
Dental calculus and dental attrition	1/32 (3.1%)
Dental calculus and tooth fracture	4/32 (12.5%)
Dental calculus, tooth fracture, and dental attrition	2/32 (6.2%)
Dental calculus, gingival recession, and dental attrition	1/32 (3.1%)
Dental calculus, gingival recession, tooth fracture, and dental attrition	1/32 (3.1%)
Dental calculus, gingival recession, periodontal disease, tooth fracture, and dental attrition	1/32 (3.1%)
Tooth fracture and dental attrition	1/32 (3.1%)
Dental attrition	1/32 (3.1%)

**Table 2:** Age, weight, and results obtained per individual. AT: absent teeth; DC: dental calculus; GR: gingival recession; PD: periodontal disease; TF: tooth fracture; DA: dental attrition.

Variable	Mean	SD	Median	Minimum	Maximum	Spearman’s Rho	*p*-Value
Age (years)	5.48	2.818	6	1	11		
Weight (kg)	27.78	5.807	28.5	14	40		
AT/individual	0.72	1.276	0	0	4	0.402	0.028
DC global index/individual	0.06	0.063	0.05	0	0.26	0.133	0.484
GR affected teeth/individual	0.84	1.648	0	0	6	0.331	0.074
PD affected teeth/individual	0.19	0.471	0	0	2	0.055	0.773
TF/individual	1.63	1.897	1	0	7	0.414	0.023
DA affected teeth/individual	4.72	6.223	1	0	21	0.551	0.002

**Table 3:** Results obtained per type of tooth. AT: absent teeth; DC: dental calculus; GR: gingival recession; PD: periodontal disease; TF: tooth fracture; DA: dental attrition. ^a,b^ Different superscript letters within rows indicate statistically significant differences among teeth (*p *< 0.05).

Variable	Grade/Type	Frequency (%)				
		Total	Incisors	Canines	Premolars	Molars
AT		23/1344 (1.7%)	11/384 (2.9%) ^a^	1/128 (0.8%) ^a,b^	11/512 (2.1%) ^a^	0/320 (0%) ^b^
DC						
	0	1248/1321 (94.5%)	369/373 (98.9%) ^a^	73/127 (57.5%) ^b^	496/501 (99.0%) ^a^	310/320 (96.9%) ^a^
	1	69/1321 (5.3%)	4/373 (1.1%) ^a^	54/127 (42.5%) ^b^	3/501 (0.6%) ^a^	8/320 (2.5%) ^a^
	2	2/1321 (0.1%)	0/373 (0%)	0/127 (0%)	1/501 (0.2%)	1/320 (0.3%)
	3	2/1321 (0.1%)	0/373 (0%)	0/127 (0%)	1/501 (0.2%)	1/320 (0.3%)
GR		27/1321 (2%)	18/373 (4.8%) ^a^	1/127 (0.8%) ^a,b^	3/501 (0.6%) ^b^	5/320 (1.6%) ^a,b^
PD		6/1321 (0.5%)	1/373 (0.3%)	0/127 (0%)	2/501 (0.4%)	3/320 (0.9%)
TF						
	3	25/1321 (1.9%)	4/373 (1.1%) ^a^	16/127 (12.6%) ^b^	5/501 (1.0%) ^a^	0/320 (0%) ^a^
	4	27/1321 (2%)	7/373 (1.9%) ^a^	5/127 (3.9%) ^b^	12/501 (2.45) ^a^	3/320 (0.9%) ^a^
DA		151/1321 (11.4%)	117/373 (31.4%) ^a^	4/127 (3.1%) ^b^	24/501 (4.8%) ^b^	6/320 (1.9%) ^b^

## Data Availability

Data supporting reported results can be sent to anyone interested by contacting the corresponding author.
